# Recurrent disc herniation following percutaneous endoscopic lumbar discectomy preferentially occurs when Modic changes are present

**DOI:** 10.1186/s13018-020-01695-6

**Published:** 2020-05-14

**Authors:** Lu Hao, Shengwen Li, Junhui Liu, Zhi Shan, Shunwu Fan, Fengdong Zhao

**Affiliations:** 1 Department of Orthopaedics, Sir Run Run Shaw Hospital, School of Medicine, Key Laboratory of Musculoskeletal System Degeneration and Regeneration Translational Research of Zhejiang Province, Zhejiang University, No. 3, Qingchun Road East, Hangzhou, 310016 People’s Republic of China; 2 Department of Orthopaedics, Haining County People’s Hospital, No.2 Qianjiangxi Road, Haining, People’s Republic of China

**Keywords:** Modic changes, Recurrent disc herniation, Percutaneous endoscopic lumbar discectomy, Herniated cartilage

## Abstract

**Objective:**

To investigate the relationship between Modic changes (MCs) and recurrent lumbar disc herniation (rLDH) and that between the herniated disc component and rLDH following percutaneous endoscopic lumbar discectomy (PELD).

**Methods:**

We included 102 (65 males, 37 females, aged 20–66 years) inpatients who underwent PELD from August 2013 to August 2016. All patients underwent CT and MRI preoperatively. The presence and type of Modic changes were assessed. During surgery, the herniated disc component of each patient was classified into two groups: nucleus pulposus group and hyaline cartilage group. The association of herniated disc component with Modic changes was investigated. The incidence of rLDH was assessed based on a more than 2-year follow-up.

**Results:**

In total, 11 patients were lost to follow-up; the other 91 were followed up during 24–60 months. Of the 91 patients, 99 discs underwent PELD; 28/99 (28.3%) had MCs. Type I and II MCs were seen in 9 (9.1%) and 19 (19.2%), respectively; no type III MCs were found. Among 28 endplates with MCs, according to the herniated disc component, 18/28 (64.3%) showed evidence of hyaline cartilage in the intraoperative specimens, including 6/9 and 12/19 endplates with type I and II MCs, respectively. Among 71 endplates without MCs, 14/71 (19.7%) showed evidence of hyaline cartilage in the intraoperative specimens. Hyaline cartilage was more common in patients with MCs (*P* < 0.05). We found 2 cases of rLDH in the non-MC group (*n* = 71); 6 cases of rLDH were found in the MC group (*n* = 28), including 2 and 4 cases for types I and II, respectively. There was no significant difference between types I and II (*P* > 0.05). rLDH was more common in patients with MCs (*P* < 0.05). We found 5 rLDH cases in the hyaline cartilage group (*n* = 32); 3 rLDH cases were found in the nucleus pulposus group (*n* = 67). rLDH was more common in the hyaline cartilage group (*P* < 0.05).

**Conclusions:**

rLDH following PELD preferentially occurs when MCs or herniated cartilage are present.

## Introduction

Percutaneous endoscopic lumbar discectomy (PELD), a minimally invasive spinal procedure, has become increasingly well accepted by both surgeons and patients who suffer from lumbar disc herniation (LDH). Compared with conventional open surgery, this technique allows the removal of the herniated disc through a very small skin incision [1]. However, along with the widespread use of PELD, many researchers have stated concerns regarding recurrence [2, 3].

Recurrent lumbar disc herniation (rLDH) has been defined as disc herniation at the same level with the reappearance of the same pain after a pain-free interval and magnetic resonance imaging (MRI) confirmation. The recurrence rate of LDH has been reported to be 5–15% [3–8]. There have been many studies designed to determine the recurrence of LDH, and various risk factors were suggested including disc degeneration, trauma, age, smoking, gender, and obesity [3, 4]. Radiologically identifiable factors, such as disc degeneration, disc height, and sagittal range of motion have been shown to be related to spinal instability and consequently to rLDH [8–10].

Modic changes, which are present as signal alterations in the vertebral endplate and adjacent bone marrow, are found on T1- and T2-weighted MRI. These changes are associated with vertebral endplate fissures and disc herniation [11–13]. However, little is known about the relationship between MCs and rLDH.

MCs are known to be associated with LDH-containing cartilaginous fragments [11–13]. Schmid et al. reported the presence of a cartilaginous endplate in the extruded disc material in 63% of patients with MCs [11] and the association between the cartilage endplate and spontaneous resorption has been specifically investigated [12]. However, the relationship between LDH-containing cartilaginous fragments and rLDH has received little attention.

The purpose of this study was to investigate the relationship between MCs and rLDH and that between the herniated disc component and rLDH following PELD.

## Materials and methods

### Inclusion criteria

Patients were included if they had radicular pain for at least 3 months that was refractory to 6 weeks of conservative treatment with or without neurological deficit, numbness in the lumbar spine, buttock, and/or lower extremity, and MRI and computed tomography (CT) demonstrated anatomical LDH correlating with symptoms and no disc calcification.

### Exclusion criteria

Patients were excluded if they had any of the following: prior lumbar surgery at another institution, disc calcification, segmental instability, vertebral fractures, spinal infection, other types of degenerative disc disease, tumors, or pregnancy.

### Recurrent disc herniation

Recurrent herniation was defined as follows: (1) patients showed a successful PELD operation as confirmed by a pain-free interval of at least 1 month and (2) reappearance of the same pain as presentation and MRI confirmation of the recurrent herniation on the same level.

### CT and MRI

CT scan of the lumbar spine was performed using a 16-slice CT scanner (GE LightSpeed Pro 16; GE Healthcare) with a detector configuration of 16 × 1.25 mm. A standard lumbar spine protocol with a tube voltage of 120 kV, tube current of 100–650 mA, and a rotation time of 0.8 s was used. Automatic tube current modulation based on the patient’s size and X-ray attenuation was used. The slice thickness and reconstruction interval were 1.25 mm and 0.625 mm, respectively.

MRI images of the lumbar spine were obtained using a General Electric 1.5-T magnet with a T1-weighted sequence (repetition time/echo time, 560 ms/12 ms; field of view, 320 × 256; receiver bandwidth, variable; 4.0-mm slice with a gap of 1.0 mm; number of excitations, 3) and a T2-weighted sequence (repetition time/echo time, 3000 ms/100 ms; field of view, 320 × 256; receiver bandwidth, variable; 4.0-mm slice with a gap of 1.0 mm; number of excitations, 3).

### Patients

**Fig. 1:**
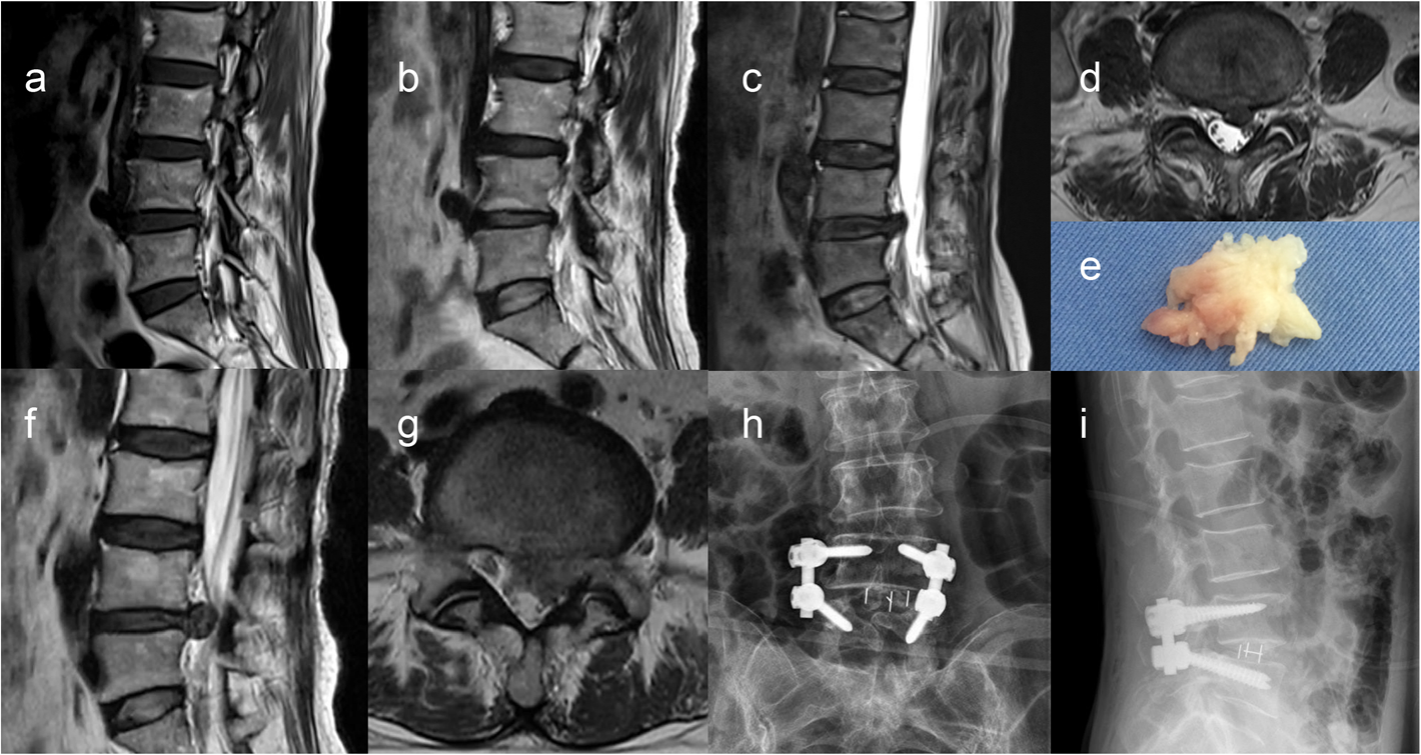
Example of rLDH on the L4/5 level in a 52 years old male with MCs: T1-weighted (**a**) and T2-weighted (**b**) MR images of lumbar endplates showed the presence of type II Modic changes (indicated by high signal on T1 and T2) on L4/5 level. MRI showed disc herniation on the same level (**c**, **d**); the herniated cartilage was present in the intraoperative specimen (**e**). Reappearance of the same pain as presentation and MRI confirmation of the recurrent herniation on the same level after 6 months follow-up (**f**, **g**), then fusion was performed (**h**, **i**)

According to the inclusion and exclusion criteria, we included the inpatients who underwent PELD from August 2013 to August 2016; the patients were consecutively enrolled. All patients had undergone CT and MRI examinations that were evaluated retrospectively. CT was used to evaluate whether the herniated disc component had a calcification. The presence, location, and type of MCs were assessed from the MRI scans. According to the herniated disc component in the intraoperative specimens, the herniated disc component of each patient was classified into two groups: the nucleus pulposus group and the hyaline cartilage group. The association of herniated disc component with MCs was investigated. Moreover, patients were also divided into the MC group and non-MC group. The incidence of rLDH was compared between the nucleus pulposus and hyaline cartilage groups and between the MC and non-MC groups during a follow-up period of at least 2 years (Fig. 1).

Ethical approval was obtained from the Medical Ethics Committee of the hospital. Additionally, all patients provided written informed consent for their information to be stored in the hospital’s database and used for research.

### Data analysis

Data were inputted into Excel (Microsoft Corp., Redmond, Washington), and quantitative results were expressed in terms of a mean and standard deviation. Data were transferred to SPSS version 20.0 (IBM Corp., Armonk, NY, USA). Group *t* tests (two-sided), and chi-square tests, were used to compare mean values, and proportions, respectively. A significance level of 0.05 was used, and *P* < 0.05 was considered significant without multiple test adjustment.

## Results

In total, 102 patients (65 males, 37 females; age, 50.9 ± 7.9 years; age range, 20–66 years) underwent PELD and were enrolled in the study; 11 patients were lost to follow-up; the other 91 patients (57 males, 34 females, age, 50.8 ± 7.8 years; age range, 20–66 years) were followed up for more than 2 years (24–60 months). Of the remaining 91 patients, 99 discs underwent PELD, and 28/99 (28.3%) had MCs; type I and II MCs were seen in 9 (9.1%) and 19 (19.2%), respectively. No type III MCs were found.

### MCs and hyaline cartilage

Among 28 endplates with MCs, according to the herniated disc component, 18/28 (64.3%) showed evidence of hyaline cartilage in the intraoperative specimens, including 6/9 endplates with type I MCs and 12/19 endplates with type II. There was no significant difference between types I and II (*P* > 0.05). Among 71 endplates without MCs, 14/71 (19.7%) showed evidence of hyaline cartilage in the intraoperative specimens. Hyaline cartilage was more common in patients with MCs (*P* < 0.05).

### MCs and rLDH

rLDH was more common in patients with MCs (*P* < 0.05) (Table 1). There were 2/71 (2.8%) cases of rLDH in the non-MC group (*n* = 71); 6/28 (21.4%) cases of rLDH were found in the MC group (*n* = 28), including 2 and 4 cases of types I and II, respectively. There was no significant difference between type I and II observations (*P* > 0.05).

**Table 1:** rLDH patient data of the MC and no-MC groups and the hyaline cartilage and nucleus pulposus groups

	rLDH	No rLDH	*P* value
MC group	6	22	0.006
Non-MC group	2	69	
Hyaline cartilage group	5	27	0.029
Nucleus pulposus group	3	64	

### Hyaline cartilage and rLDH

rLDH was more common in the hyaline cartilage group (*P* < 0.05) (Table 1). There were 5/32 (15.6%) cases of rLDH in the hyaline cartilage group (*n* = 32) and 3/67 (4.5%) cases of rLDH in the nucleus pulposus group (*n* = 67).

## Discussion

### Summary of findings

To our knowledge, this study is the first to investigate the relationship between MCs and rLDH and that between the herniated disc component and rLDH following PELD. We found that hyaline cartilage and rLDH were more common in patients with MCs. rLDH was more common in the hyaline cartilage group. rLDH following PELD preferentially occurred when MCs or the herniated cartilage are present.

### MCs and herniated cartilage

The variable composition of herniated tissue has been described previously, with various proportions of nucleus pulposus, annulus, hyaline cartilage, and bone being reported. Tanaka et al. [14] suggested that the cartilaginous endplate-osteochondral junction is weak, whereas the cartilaginous endplate-inner annulus fibrosus connection is strong, so the herniated tissue usually contains a cartilaginous endplate. Rajasekaran et al. [15] reported that a high proportion of Indian patients had herniations containing cartilage and bone.

MCs, which present as signal alterations in the vertebral endplate and adjacent bone marrow, are found on T1- and T2-weighted MRI. MCs are known to be associated with LDH-containing cartilaginous fragments [11–13]. Schmid et al. reported the presence of a cartilaginous endplate in the extruded disc material in 63% of patients with MCs. Our study confirmed the results; 64.3% of our patients with MCs showed evidence of hyaline cartilage in the intraoperative specimens, and only 19.7% showed evidence of hyaline cartilage. Hyaline cartilage was more common in patients with MCs.

### MCs and rLDH

Our study showed that rLDH preferentially occurred when MCs or the herniated cartilage were present. For patients with MCs, these conditions usually imply that the endplate structure has been damaged. The herniated cartilaginous endplate is only one part of the damaged endplate, because the connection between the cartilage endplate and the vertebral body is relatively weak, so other parts of the endplate easily separate from the vertebral body and herniate with the nucleus pulposus, and rLDH occurs. Similar to the MCs endplate, if the herniated cartilage is present in the intraoperative specimen, which also reflects damage to the endplate structure, other parts of the endplate easily separate from the vertebral body and herniate with nucleus pulposus, and rLDH preferentially occurs.

Our study has some limitations. Firstly, it was a retrospective study with a small sample size. The relatively small sample size limits the accuracy of correlation between the rate of rLDH and the type of MCs. Secondly, the study focused on the phenotypic association between rLDH and MCs or the herniated disc component, while no correlative mechanism was studied. In particular, we did not confirm the results using biomechanics and histomorphologic methods. Further study is needed in the future.

Nevertheless, this study suggests that rLDH preferentially occurs after PELD when MCs are present. If we choose PELD for patients with MCs, we need to inform these patients that they have a higher incidence risk of rLDH, especially for old patients. Meanwhile, if cartilage is present in the herniated disc component, we also should inform these patients that they have a higher incidence risk of rLDH. Such patients may require a second operation.

## Conclusions

rLDH following PELD preferentially occurred when MCs or the herniated cartilage were present. Patients with MCs had a higher incidence risk of rLDH.

## Data Availability

The datasets used and analyzed during the current study are available from the corresponding author on reasonable request.

## Abbreviations

MCs: Modic changes
rLDH: Recurrent lumbar disc herniation
PELD: Percutaneous endoscopic lumbar discectomy
